# Castration of Criminals

**Published:** 1892-06

**Authors:** 


### CASTRATION FOR CRIMINALS.

Prof. W. A. Hammond read a paper before the Washington City Medical Club in which he advocated castration as a substitute for capital punishment for crime. The paper was well received and ably discussed, pro and con. If crime be hereditary it certainly would be a good idea to stop the breed; and we venture to say the average evil doer would more dread castration than he would the gallows or the penitentiary. There is always a hope of escape from the latter, or a pardon, or a reprieve or escape, in case of death sentence; but castration? it is sure and certain, and when done cannot be undone.

The JOURNAL has always opposed capital punishment, on the grounds that it does not lessen crime, and on the score of econ omy. In a case deserving death under our laws, castrate the criminal and put him to work for the good of the community which he has outraged and forfeited,—confining him at night in the penitentiary. Dr. Hammond is right; stop the breed of criminals, and at the same time inflict a punishment which will strike more terror to the soul of other evil doers than hanging does.

